# Crystal structure, Hirshfeld surface analysis and DFT studies of 5-bromo-1-{2-[2-(2-chloro­eth­oxy)eth­oxy]eth­yl}indoline-2,3-dione

**DOI:** 10.1107/S2056989019011617

**Published:** 2019-08-30

**Authors:** Omar Abdellaoui, Tuncer Hökelek, Frédéric Capet, Catherine Renard, Amal Haoudi, Mohamed Khalid Skalli, Youssef Kandri Rodi

**Affiliations:** a Laboratoire de Chimie Organique Appliquée, Université Sidi Mohamed Ben Abdallah, Faculté des Sciences et Techniques, Route d’immouzzer, BP 2202, Fez, Morocco; b Department of Physics, Hacettepe University, 06800 Beytepe, Ankara, Turkey; c Univ. Lille, CNRS, Centrale Lille, ENSCL, Univ. Artois, UMR 8181–UCCS–Unité de Catalyse et Chimie du Solide, F-59000 Lille, France

**Keywords:** crystal structure, bromo­indoline, dione, π-stacking, DFT, Hirshfeld surface

## Abstract

The title compound, C_14_H_15_BrClNO_4_, consists of a 5-bromo­indoline-2,3-dione unit linked to a 1-{2-[2-(2-chloro­eth­oxy)eth­oxy]eth­yl} moiety. In the crystal, a series of C—H⋯O hydrogen bonds link the molecules to form a supramolecular three-dimensional structure, enclosing *R*_2_^2^(8), *R*_2_^2^(12), *R*_2_^2^(18) and *R*_2_^2^(22) ring motifs. π–π contacts between the five-membered dione rings may further stabilize the structure, with a centroid–centroid distance of 3.899 (2) Å. The Hirshfeld surface analysis of the crystal structure indicates that the most important contributions for the crystal packing are from H⋯H (28.1%), H⋯O/O⋯H (23.5%), H⋯Br/Br⋯H (13.8%), H⋯Cl/Cl⋯H (13.0%) and H⋯C/C⋯H (10.2%) inter­actions. Hydrogen bonding and van der Waals inter­actions are the dominant inter­actions in the crystal packing. Density functional theory (DFT) optimized structures at the B3LYP/6-311G(d,p) level are compared with the experimentally determined mol­ecular structure in the solid state. The HOMO—LUMO behaviour was elucidated to determine the energy gap. The chloro­eth­oxy­ethoxyethyl side chain atoms are disordered over two sets of sites with an occupancy ratio of 0.665 (8):0.335 (6).

## Abstract 2

The title compound consists of the 5-bromo­indoline-2,3-dione unit linked by a 1-{2-[2-(2 chloro­eth­oxy)eth­oxy]eth­yl} moiety. In the crystal, inter­molecular C—H_Brmind_⋯O_Dio_, C—H_Brmind_⋯O_Ethy_, C—H_Chlethy_⋯O_Dio_ and C—H_Chlethy_⋯O_Chlethy_ (Brmind = bromo­indoline, Dio = dione, Ethy = eth­oxy and Chlethy = chloro­eth­oxy) hydrogen bonds link the mol­ecules into a three-dimensional structure, enclosing 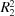
(8), 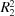
(12), 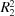
(18) and 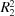
(22) ring motifs. The π–π contacts between the five-membered dione rings may further stabilize the structure.

## Chemical context   

Heterocycles are a class of chemical compounds in which one atom or more than one carboxyl group is replaced by a heteroatom such as oxygen, nitro­gen, phospho­rus or sulfur. They are very inter­esting chemical compounds because of their potential applications in different fields. The most common heterocycles contain nitro­gen and oxygen (Pathak & Bahel, 1980 ▸; Naik & Malik, 2010 ▸; Srivalli *et al.*, 2011 ▸). The chemistry of nitro­gen compounds is the preferred source for a large number of study subjects in the laboratory. The N atom is present in several natural mol­ecules of pharmacological inter­est, so many methods have been developed to access nitro­gen compounds, especially heterocyclic compounds. Given the biological inter­est of heterocyclic compounds, we have been inter­ested in synthesizing new polyfunctional heterocyclic systems capable of presenting potential applications. The chemistry of isatin is already well documented due to its wide range of applications, especially in organic synthetic chemistry and medicinal chemistry. The first reports on the syntheses of isatin and isatin-based derivatives can be traced back to the first half of the 19th century, and almost one hundred years after those publications, the review ‘The Chemistry of Isatin’ showed the versatility of this mol­ecular fragment. This reaction is also used for the synthesis of natural products, such as sugar derivatives (DeShong *et al.*, 1986 ▸), β-lactams (Kametani *et al.*, 1988 ▸), amino acids (Annuziata *et al.*, 1986) and alkaloids (Asrof Ali *et al.*, 1988 ▸), and products with pharmacological inter­est, such as pyrazolines, which have several biological activities (Araino *et al.*, 1996 ▸; Harrison *et al.*, 1996 ▸). As a continuation of our research devoted to the development of substituted 5-bromo­indoline-2,3-dione derivatives, we report herein the synthesis and mol­ecular and crystal structures, along with the Hirshfeld surface analysis and the density functional theory (DFT) computational calculations carried out at the B3LYP/6-311G(d,p) level, of a 5-bromo­indoline-2,3-dione derivative by the alkyl­ation reaction of 5-bromo-1*H*-indole-2,3-dione under phase-transfer catalysis conditions using tetra-*n*-butyl­ammonium bromide (TBAB) as catalyst and potassium carbonate as base, leading to the title compound, (I).
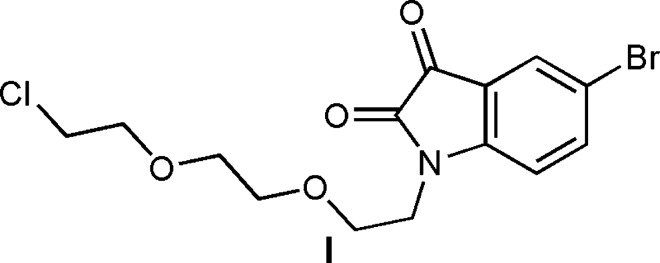


## Structural commentary   

**Figure 1:**
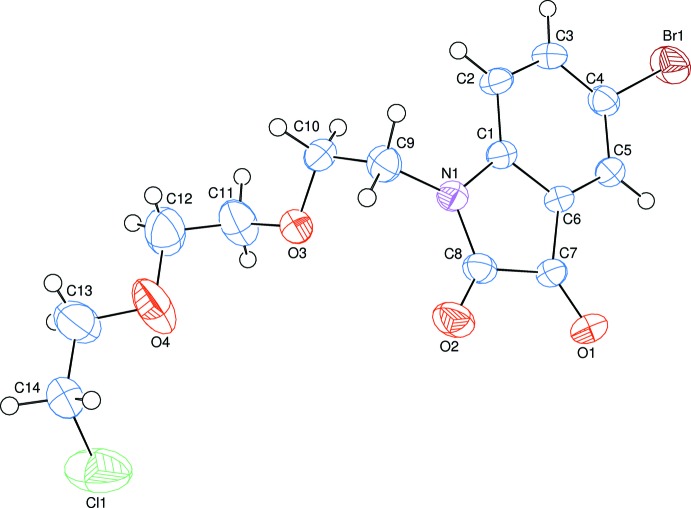
The mol­ecular structure of the title compound with the atom-numbering scheme. Displacement ellipsoids are drawn at the 50% probability level. Only the major component of disorder is shown for clarity.

The title compound, (I), consists of an 5-bromo­indoline-2,3-dione unit linked to a 1-{2-[2-(2-chloro­eth­oxy)eth­oxy]eth­yl} moiety (Fig. 1 ▸). The planar six- and five-membered benzene and dione rings, *i.e.* *A* (atoms C1–C6) and *B* (N1/C1/C6–C8), are oriented at a dihedral angle of *A*/*B* = 2.78 (6)°. Atoms Br1, O1 and C9 are at distances of 0.0415 (4), 0.0464 (8) and −0.0244 (7) Å, respectively, from the best plane of the bromo­indoline unit. The 1-{2-[2-(2-chloro­eth­oxy)eth­oxy]eth­yl} moiety is oriented with respect to the bromo­indoline unit by 77.7 (2)°, as defined by the C10—C9—N1—C1 torsion angle.

## Supra­molecular features   

**Figure 2:**
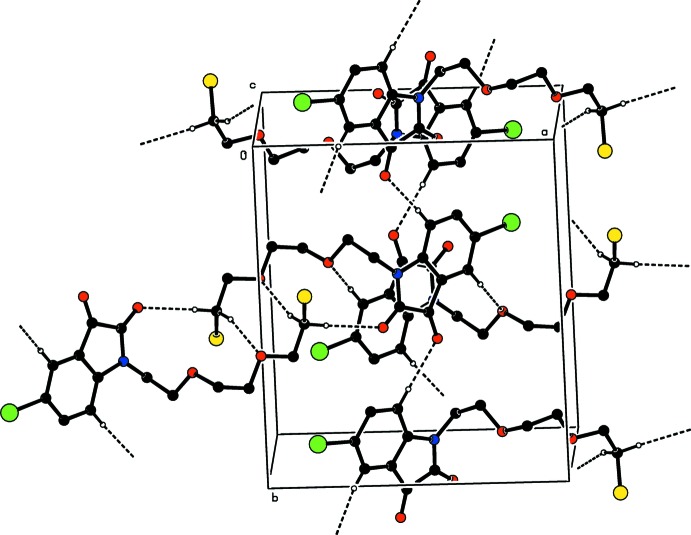
A partial packing diagram, viewed down the *c*-axis direction. C—H_Brmind_⋯O_Dio_, C—H_Brmind_⋯O_Ethy_, C—H_Chlethy_⋯O_Dio_ and C—H_Chlethy_⋯O_Chlethy_ (Brmind = bromo­indoline, Dio = dione, Ethy = eth­oxy and Chlethy = chloro­eth­oxy) hydrogen bonds are indicated by dashed lines. Only the major component of disorder is shown for clarity.

In the crystal, inter­molecular C—H_Brmind_⋯O_Dio_, C—H_Brmind_⋯O_Ethy_, C—H_Chlethy_⋯O_Dio_ and C—H_Chlethy_⋯O_Chlethy_ (Brmind = bromo­indoline, Dio = dione, Ethy = eth­oxy and Chlethy = chloro­eth­oxy) hydrogen bonds (Table 1 ▸) link the mol­ecules into a three-dimensional structure, enclosing 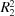
(8), 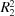
(12), 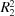
(18) and 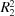
(22) ring motifs (Fig. 2 ▸). π–π contacts between the five-membered rings, *Cg*1—*Cg*1^i^ [symmetry code: (i) −*x* + 1, −*y* + 1, −*z* + 1, where *Cg*1 is the centroid of ring *A* (atoms N1/C1/C6–C8)], may further stabilize the structure, with a centroid–centroid distance of 3.899 (2) Å. The Hirshfeld surface analysis of the crystal structure indicates that the most important contributions for the crystal packing are from H⋯H (28.1%), H⋯O/O⋯H (23.5%), H⋯Br/Br⋯H (13.8%), H⋯Cl/Cl⋯H (13.0%) and H⋯C/C⋯H (10.2%) inter­actions. Hydrogen bonding and van der Waals inter­actions are the dominant inter­actions in the crystal packing.

## Hirshfeld surface analysis   

**Figure 3:**
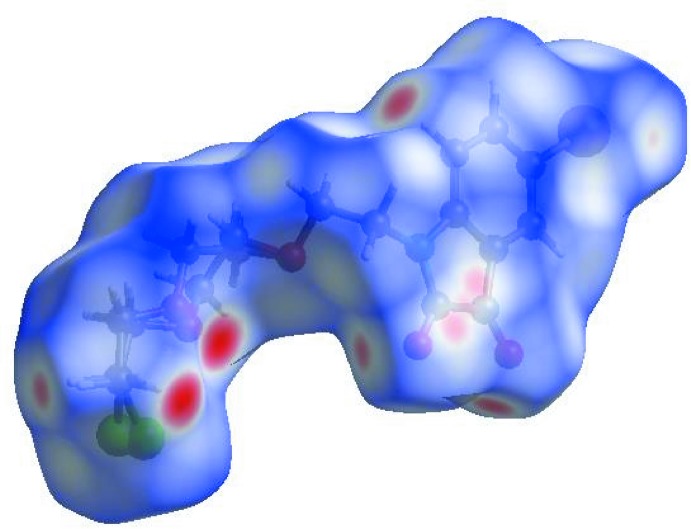
View of the three-dimensional Hirshfeld surface of the title compound plotted over *d*_norm_ in the range −0.3481 to 1.0316 a.u.

**Figure 4:**
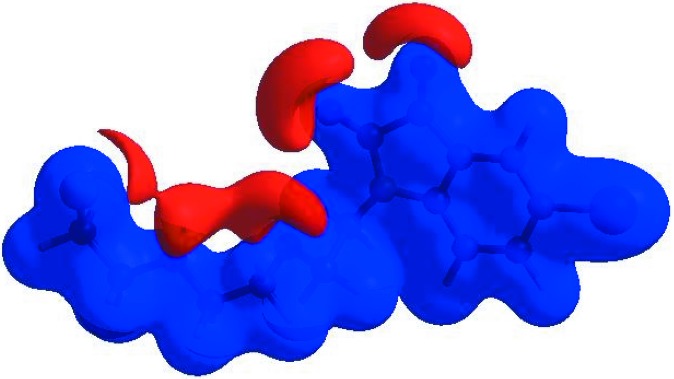
View of the three-dimensional Hirshfeld surface of the title compound plotted over electrostatic potential energy in the range −0.0500 to 0.0500 a.u. using the STO-3G basis set at the Hartree–Fock level of theory. Hydrogen-bond donors and acceptors are shown as blue and red regions around the atoms corresponding to positive and negative potentials, respectively.

**Figure 5:**
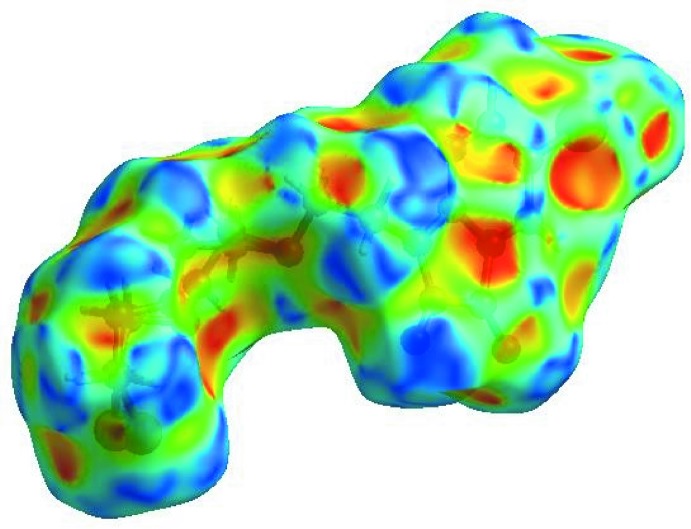
Hirshfeld surface of the title compound plotted over shape-index.

**Figure 6:**
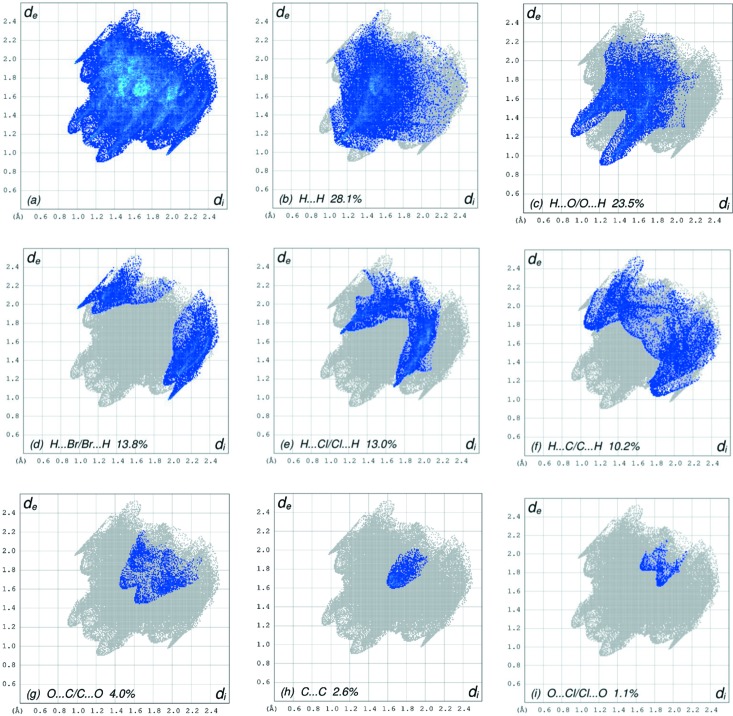
The full two-dimensional fingerprint plots for the title compound, showing (*a*) all inter­actions, and delineated into (*b*) H⋯H, (*c*) H⋯O/O⋯H, (*d*) H⋯Br/Br⋯H, (*e*) H⋯Cl/Cl⋯H, (*f*) H⋯C/C⋯H, (*g*) O⋯C/C⋯O, (*h*) C⋯C and (*i*) O⋯Cl/Cl⋯O inter­actions. The *d*_i_ and *d*_e_ values are the closest inter­nal and external distances (in Å) from given points on the Hirshfeld surface contacts.

In order to visualize the inter­molecular inter­actions in the crystal of the title compound, a Hirshfeld surface (HS) analysis (Hirshfeld, 1977 ▸; Spackman & Jayatilaka, 2009 ▸) was carried out using *CrystalExplorer17.5* (Turner *et al.*, 2017 ▸). In the HS plotted over *d*_norm_ (Fig. 3 ▸), the white surface indicates contacts with distances equal to the sum of the van der Waals radii, and the red and blue colours indicate distances shorter (in close contact) or longer (distinct contact) than the van der Waals radii, respectively (Venkatesan *et al.*, 2016 ▸). The bright-red spots appearing near atoms O1, O2 and O4, and H atoms H2, H14*A* and H14*B*, indicate their roles as the respective donors and/or acceptors; they also appear as blue and red regions corresponding to positive and negative potentials on the HS mapped over electrostatic potential (Spackman *et al.*, 2008 ▸; Jayatilaka *et al.*, 2005 ▸), as shown in Fig. 4 ▸. The blue regions indicate the positive electrostatic potential (hydrogen-bond donors), while the red regions indicate the negative electrostatic potential (hydrogen-bond acceptors). The shape-index of the HS is a tool to visualize the π–π stacking by the presence of adjacent red and blue triangles; if there are no adjacent red and/or blue triangles, then there are no π–π inter­actions. Fig. 5 ▸ clearly suggests that there is a π–π inter­action in (I). The overall two-dimensional fingerprint plot (Fig. 6 ▸ *a*) and those delineated into H⋯H, H⋯O/O⋯H, H⋯Br/Br⋯H, H⋯Cl/Cl⋯H, H⋯C/C⋯H, O⋯C/C⋯O, C⋯C and O⋯Cl/Cl⋯O contacts (McKinnon *et al.*, 2007 ▸) are illustrated in Figs. 6 ▸(*b*)–(*i*), respectively, together with their relative contributions to the Hirshfeld surface. The most important inter­action is H⋯H, contributing 28.1% to the overall crystal packing, which is reflected in Fig. 6 ▸(*b*) as widely scattered points of high density due to the large hydrogen content of the mol­ecule with the tip at *d*_e_ = *d*_i_ ∼1.08 Å, due to the short inter­atomic H⋯H contacts (Table 2 ▸). The pair of characteristic wings resulting in the fingerprint plot delineated into H⋯O/O⋯H contacts (Fig. 6 ▸ *c*), with a 23.5% contribution to the HS, arises from the H⋯O/O⋯H contacts (Table 2 ▸) and is viewed as a pair of spikes with the tips at *d*_e_ + *d*_i_ = 2.10 Å. The pairs of scattered points of wings resulting in the fingerprint plots delineated into H⋯Br/Br⋯H (Fig. 6 ▸ *d*) and H⋯Cl/Cl⋯H (Fig. 6 ▸ *e*) contacts, with 13.8 and 13.0% contributions to the HS, have nearly symmetrical distributions of points with the edges at *d*_e_ + *d*_i_ = 2.92 (for thin edge) and 3.20 Å (for thick edge) and *d*_e_ + *d*_i_ = 2.78 Å, respectively, arising from the H⋯Br/Br⋯H and H⋯Cl/Cl⋯H contacts (Table 2 ▸). In the absence of C—H⋯π inter­actions, with a pair of characteristic wings resulting in the fingerprint plot delineated into H⋯C/C⋯H contacts (Fig. 6 ▸ *f*), a 10.2% contribution to the HS, arises from the H⋯C/C⋯H contacts (Table 2 ▸) and is seen as a thick pair of spikes with the tips at *d*_e_ + *d*_i_ = 2.82 Å. The pair of characteristic wings resulting in the fingerprint plot delineated into O⋯C/C⋯O contacts (Fig. 6 ▸ *g*), with a 4.0% contribution to the HS, arises from the O⋯C/C⋯O contacts (Table 2 ▸) and is seen as a pair of spikes with the tips at *d*_e_ + *d*_i_ = 3.05 Å. The C⋯C contacts (Fig. 6 ▸ *h*), with a 2.6% contribution to the HS, have a nearly arrow-shaped distribution of points arising from the C⋯C contacts (Table 2 ▸) and is seen with the tip at *d*_e_ = *d*_i_ ∼1.62 Å. Finally, the pair of scattered points of wings resulting in the fingerprint plot delineated into O⋯Cl/Cl⋯O (Fig. 6 ▸ *i*) contacts, with a 1.1% contribution to the HS, have nearly symmetrical distributions of points with the edge at *d*_e_ + *d*_i_ = 3.50 Å.

**Figure 7:**
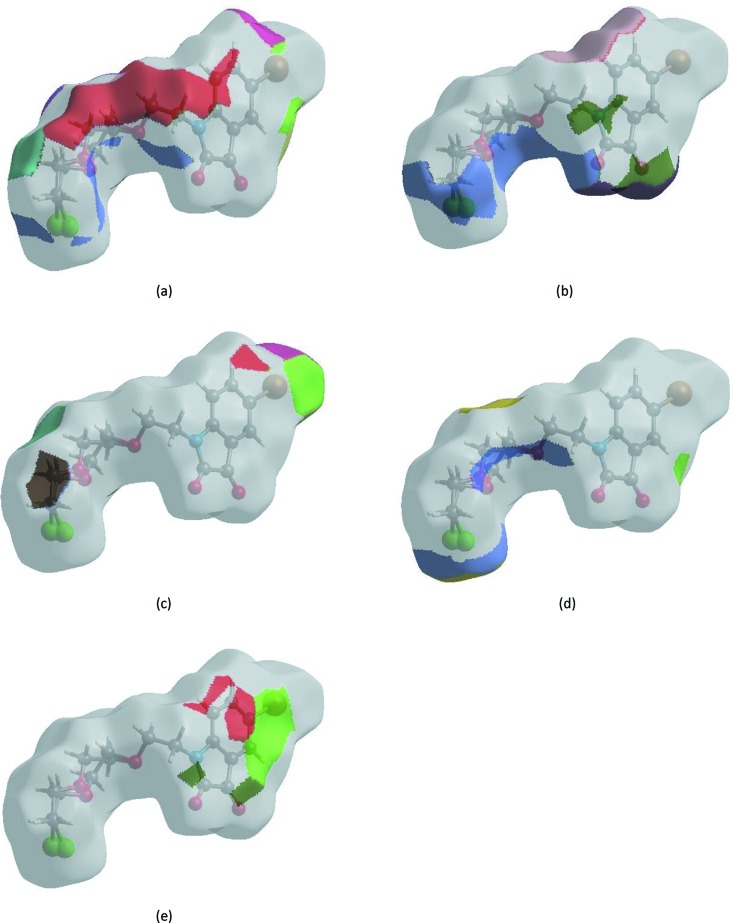
The Hirshfeld surface representations with the function *d*_norm_ plotted onto the surface for (*a*) H⋯H, (*b*) H⋯O/O⋯H, (*c*) H⋯Br/Br⋯H, (*d*) H⋯Cl/Cl⋯H and (*e*) H⋯C/C⋯H inter­actions.

The Hirshfeld surface representations with the function *d*_norm_ plotted onto the surface are shown for the H⋯H, H⋯O/O⋯H, H⋯Br/Br⋯H, H⋯Cl/Cl⋯H and H⋯C/C⋯H inter­actions in Figs. 7 ▸(*a*)–(*e*), respectively.

The Hirshfeld surface analysis confirms the importance of H-atom contacts in establishing the packing. The large number of H⋯H, H⋯C/C⋯H and H⋯O/O⋯H inter­actions suggest that van der Waals inter­actions and hydrogen bonding play the major roles in the crystal packing (Hathwar *et al.*, 2015 ▸).

## DFT calculations   

**Figure 8:**
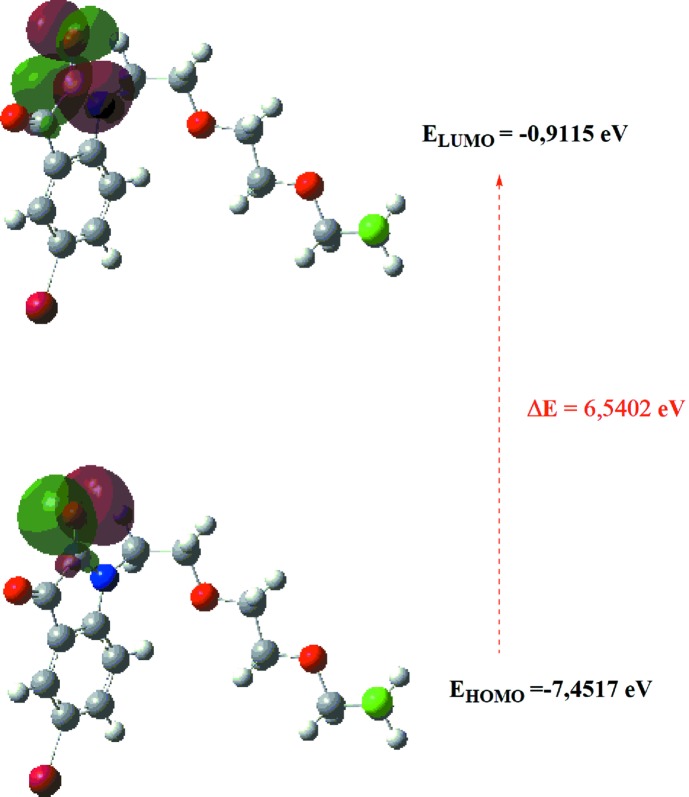
The energy band gap of the title compound.

The optimized structure of the title compound, (I), in the gas phase was generated theoretically *via* density functional theory (DFT) using standard B3LYP functional and 6-311G(d,p) basis-set calculations (Becke, 1993 ▸), as implemented in *GAUSSIAN09* (Frisch *et al.*, 2009 ▸). The theoretical and experimental results were in good agreement (Table 4). The highest-occupied mol­ecular orbital (HOMO), acting as an electron donor, and the lowest-unoccupied mol­ecular orbital (LUMO), acting as an electron acceptor, are very important parameters for quantum chemistry. When the energy gap is small, the mol­ecule is highly polarizable and has high chemical reactivity. The electron transition from the HOMO to the LUMO energy level is shown in Fig. 8 ▸. The HOMO and LUMO are localized in the plane extending from the whole 1-{2-[2-(2-chloro­eth­oxy)eth­oxy]eth­yl}-5-bromo­indoline-2,3-dione ring. The energy band gap (Δ*E* = *E*_LUMO_ − *E*_HOMO_) of the mol­ecule was about 6.5402 eV, and the frontier mol­ecular orbital energies, *i.e.* *E*_HOMO_ and *E*_LUMO_, were −7.4517 and −0.9115 eV, respectively.

## Database survey   

A non-alkyl­ated analogue, namely 5-chloro­indoline-2,3-dione has been reported (Wei *et al.*, 2010 ▸), as well as three similar structures, namely 1-tetra­decyl­indoline-2,3-dione (Mamari *et al.*, 2010 ▸), 5-fluoro-1-(prop-2-en-1-yl)-2,3-di­hydro-1*H*-indole-2,3-dione (Qachchachi *et al.*, 2017 ▸) and 1-(morpholino­meth­yl)indoline-2,3-dione (Tang *et al.*, 2010 ▸).

## Synthesis and crystallization   

1,2-Bis(2-chloro­eth­oxy)ethane (0.26 ml, 1.86 mmol) was added dropwise to a solution of 5-bromo-1*H*-indole-2,3-dione (0.4 g, 1.76 mmol) and di­methyl­formamide (DMF, 20 ml) in potassium carbonate (0.6 g, 4.4 mmol) and tetra-*n*-butyl­ammonium bromide (0.1 g, 4.4 mmol). The mixture was stirred at 353 K for 48 h. The reaction was controlled by CCM. The solution was filtered and the DMF was removed under vacuum. The product obtained was separated by chromatography on a column of silica gel with hexa­ne–ethyl acetate (4:1 *v*/*v*) as eluent. The isolated solid was recrystallized from ethanol to afford red crystals (yield 48%, m.p. 349 K).

## Refinement   

The experimental details, including the crystal data, data collection and refinement, are summarized in Table 3 ▸. H atoms were positioned geometrically, with C—H = 0.93 and 0.97 Å for aromatic and methyl­ene H atoms, respectively, and constrained to ride on their parent atoms, with *U*_iso_(H) = 1.2*U*_eq_(C). During the refinement process, the disordered chloro­eth­oxy­ethoxyethyl side-chain atoms were refined with a major–minor occupancy ratio of 0.665 (8):0.335 (6).

## Supplementary Material

Crystal structure: contains datablock(s) I, global. DOI: 10.1107/S2056989019011617/lh5913sup1.cif

Structure factors: contains datablock(s) I. DOI: 10.1107/S2056989019011617/lh5913Isup2.hkl

Supporting information file. DOI: 10.1107/S2056989019011617/lh5913Isup3.cdx

CCDC reference: 1948316

Additional supporting information:  crystallographic information; 3D view; checkCIF report

## Figures and Tables

**Table 1:** Hydrogen-bond geometry (Å, °)

*D*—H⋯*A*	*D*—H	H⋯*A*	*D*⋯*A*	*D*—H⋯*A*
C2—H2⋯O1^vii^	0.93	2.47	3.392 (4)	174
C5—H5⋯O3^v^	0.93	2.47	3.352 (4)	158
C14—H14*A*⋯O2^iii^	0.97	2.50	3.455 (5)	168
C14—H14*B*⋯O4^iii^	0.97	2.38	3.302 (6)	161

Symmetry codes: (iii) 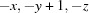; (v) 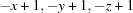; (vii) 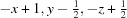.

**Table 2:** Summary of short interatomic contacts (Å)

Br1⋯H12*C* ^i^	3.05	O4⋯H14*B* ^iii^	2.38
Cl1⋯O4	3.188 (5)	O4*A*⋯H14*D* ^iii^	2.48
Cl1*A*⋯C11*A* ^ii^	3.553 (4)	C1⋯C7^v^	3.542 (4)
Cl1*A*⋯O4*A*	2.720 (14)	C2⋯C10	3.537 (4)
Cl1⋯H9*B* ^iii^	2.92	C5⋯C7^v^	3.356 (5)
Cl1*A*⋯H11*D* ^ii^	2.99	C5⋯C8^v^	3.290 (2)
O1⋯C2^iv^	3.392 (4)	C6⋯C7^v^	3.251 (4)
O1⋯O2	2.951 (4)	C6⋯C6^v^	3.327 (2)
O1⋯C10^iv^	3.294 (5)	C8⋯C8^vi^	3.324 (4)
O1⋯C1^v^	3.397 (4)	C10⋯C2	3.537 (4)
O2⋯C8^vi^	3.093 (4)	C12*A*⋯O3	2.355 (5)
O2⋯N1^vi^	3.191 (2)	C2⋯H9*A*	2.89
O3⋯C5^v^	3.352 (5)	C4⋯H13*C* ^i^	2.93
O3⋯C14^iii^	3.415 (4)	C5⋯H13*C* ^i^	2.89
O3⋯O4	2.953 (4)	C9⋯H2	2.86
O3⋯N1	2.949 (5)	C10⋯H12*A*	2.99
O3*A*⋯C5^v^	3.352 (4)	C11⋯H5^v^	2.84
O3*A*⋯N1	2.949 (5)	H2⋯H9*A*	2.48
O3*A*⋯O4*A*	2.866 (4)	H2⋯H10*B*	2.59
O4⋯C14^iii^	3.302 (4)	H5⋯H12*D* ^v^	2.58
O4*A*⋯C14*A* ^iii^	3.37 (2)	H5⋯H11*A* ^v^	2.41
O1⋯H2^iv^	2.47	H10*A*⋯H12*A*	2.52
O1⋯H9*A* ^iv^	2.77	H10*A*⋯H11*D*	2.09
O2⋯H9*B*	2.60	H10*B*⋯H11*B*	2.35
O2⋯H3^iv^	2.85	H10*B*⋯H11*C*	2.34
O2⋯H14*C* ^iii^	2.66	H12*A*⋯H13*B*	2.53
O2⋯H14*A* ^iii^	2.50	H12*B*⋯H13*A*	2.39
O3⋯H5^v^	2.47	H12*C*⋯H13*C*	2.07
O3*A*⋯H5^v^	2.47	H14*B*⋯H14*B* ^iii^	2.37

Symmetry codes: (i) 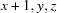; (ii) 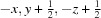; (iii) 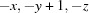; (iv) 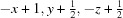; (v) 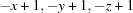; (vi) 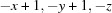.

**Table 3:** Experimental details

Crystal data	
Chemical formula	C_14_H_15_BrClNO_4_
*M*_r_	376.63
Crystal system, space group	Monoclinic, *P*2_1_/*c*
Temperature (K)	300
*a*, *b*, *c* (Å)	12.4682 (4), 14.6397 (5), 8.3524 (3)
β (°)	91.392 (2)
*V* (Å^3^)	1524.12 (9)
*Z*	4
Radiation type	Mo *K*α
μ (mm^−1^)	2.89
Crystal size (mm)	0.25 × 0.22 × 0.07

Data collection	
Diffractometer	Bruker APEXII CCD
Absorption correction	Multi-scan (*SADABS*; Bruker, 2016 ▸)
*T*_min_, *T*_max_	0.573, 0.746
No. of measured, independent and observed [*I* > 2σ(*I*)] reflections	36235, 4608, 3442
*R*_int_	0.030
(sin θ/λ)_max_ (Å^−1^)	0.713

Refinement	
*R*[*F* ^2^ > 2σ(*F* ^2^)], *wR*(*F* ^2^), *S*	0.038, 0.099, 1.04
No. of reflections	4608
No. of parameters	245
No. of restraints	11
H-atom treatment	H-atom parameters constrained
Δρ_max_, Δρ_min_ (e Å^−3^)	0.77, −0.69

Computer programs: *APEX3* and *SAINT* (Bruker, 2016 ▸), *SHELXT* (Sheldrick, 2015*a* ▸), *SHELXL2018* (Sheldrick, 2015*b* ▸) and *OLEX2* (Dolomanov *et al.*, 2009 ▸).

## supplementary crystallographic information

### Crystal data

**Table:**

C_14_H_15_BrClNO_4_	*F*(000) = 760
*M**_r_* = 376.63	*D*_x_ = 1.641 Mg m^−^^3^
Monoclinic, *P*2_1_/*c*	Mo *K*α radiation, λ = 0.71073 Å
*a* = 12.4682 (4) Å	Cell parameters from 9939 reflections
*b* = 14.6397 (5) Å	θ = 2.8–26.1°
*c* = 8.3524 (3) Å	µ = 2.89 mm^−^^1^
β = 91.392 (2)°	*T* = 300 K
*V* = 1524.12 (9) Å^3^	Plate, red
*Z* = 4	0.25 × 0.22 × 0.07 mm

### Data collection

**Table:**

Bruker APEXII CCD diffractometer	3442 reflections with *I* > 2σ(*I*)
φ and ω scans	*R*_int_ = 0.030
Absorption correction: multi-scan (SADABS; Bruker, 2016)	θ_max_ = 30.5°, θ_min_ = 1.6°
*T*_min_ = 0.573, *T*_max_ = 0.746	*h* = −17→17
36235 measured reflections	*k* = −20→20
4608 independent reflections	*l* = −11→11

### Refinement

**Table:**

Refinement on *F*^2^	Primary atom site location: dual
Least-squares matrix: full	Secondary atom site location: difference Fourier map
*R*[*F*^2^ > 2σ(*F*^2^)] = 0.038	Hydrogen site location: inferred from neighbouring sites
*wR*(*F*^2^) = 0.099	H-atom parameters constrained
*S* = 1.04	*w* = 1/[σ^2^(*F*_o_^2^) + (0.0412*P*)^2^ + 0.8377*P*] where *P* = (*F*_o_^2^ + 2*F*_c_^2^)/3
4608 reflections	(Δ/σ)_max_ < 0.001
245 parameters	Δρ_max_ = 0.77 e Å^−^^3^
11 restraints	Δρ_min_ = −0.69 e Å^−^^3^

### Special details

**Table:**

Geometry. All esds (except the esd in the dihedral angle between two l.s. planes) are estimated using the full covariance matrix. The cell esds are taken into account individually in the estimation of esds in distances, angles and torsion angles; correlations between esds in cell parameters are only used when they are defined by crystal symmetry. An approximate (isotropic) treatment of cell esds is used for estimating esds involving l.s. planes.
Refinement. At the end of the refinement, it remained some residual electronic density pics around O4 and C12. suggesting a disorder. We modeled this disorder considering two positions with following occupancies : 0.665 (7) and 0.335 (7). The R1(Fo > 4sig(Fo)) factor decreased from 5.96% to 3.76%.

### Fractional atomic coordinates and isotropic or equivalent isotropic displacement parameters (Å^2^)

**Table:**

	*x*	*y*	*z*	*U*_iso_*/*U*_eq_	Occ. (<1)
Br1	0.82107 (2)	0.34602 (2)	0.67781 (3)	0.06075 (11)	
O1	0.57179 (13)	0.62280 (10)	0.3141 (2)	0.0521 (4)	
O2	0.40213 (12)	0.55268 (11)	0.09446 (18)	0.0497 (4)	
N1	0.45304 (12)	0.41469 (10)	0.21008 (18)	0.0339 (3)	
C1	0.53610 (14)	0.38646 (12)	0.3166 (2)	0.0308 (3)	
C2	0.56401 (15)	0.29844 (12)	0.3613 (2)	0.0355 (4)	
H2	0.526977	0.248176	0.320193	0.043*	
C3	0.64948 (16)	0.28779 (13)	0.4700 (2)	0.0380 (4)	
H3	0.670281	0.229390	0.501726	0.046*	
C4	0.70420 (15)	0.36307 (13)	0.5319 (2)	0.0380 (4)	
C5	0.67705 (15)	0.45129 (13)	0.4869 (2)	0.0366 (4)	
H5	0.713826	0.501486	0.528804	0.044*	
C6	0.59299 (14)	0.46176 (12)	0.3772 (2)	0.0320 (3)	
C7	0.54778 (15)	0.54334 (12)	0.3003 (2)	0.0364 (4)	
C8	0.45658 (15)	0.50726 (13)	0.1861 (2)	0.0364 (4)	
C9	0.38069 (16)	0.35247 (14)	0.1244 (2)	0.0410 (4)	
H9A	0.422101	0.302168	0.082394	0.049*	
H9B	0.347871	0.384527	0.034163	0.049*	
C10	0.29340 (17)	0.31449 (14)	0.2271 (3)	0.0439 (4)	
H10A	0.253843	0.267628	0.168553	0.053*	0.665 (6)
H10B	0.324814	0.287076	0.322966	0.053*	0.665 (6)
H10C	0.253843	0.267628	0.168553	0.053*	0.335 (6)
H10D	0.324814	0.287076	0.322966	0.053*	0.335 (6)
Cl1	−0.1661 (2)	0.5843 (2)	0.1862 (3)	0.0789 (6)	0.665 (6)
O3	0.22328 (11)	0.38602 (10)	0.26936 (19)	0.0453 (3)	0.665 (6)
O4	−0.0082 (4)	0.4132 (3)	0.2056 (7)	0.0581 (10)	0.665 (6)
C11	0.1364 (4)	0.3629 (5)	0.3717 (7)	0.0464 (14)	0.665 (6)
H11A	0.120622	0.414886	0.439200	0.056*	0.665 (6)
H11B	0.158212	0.312658	0.440759	0.056*	0.665 (6)
C12	0.0374 (3)	0.3365 (2)	0.2790 (5)	0.0488 (10)	0.665 (6)
H12A	0.055106	0.291674	0.198247	0.059*	0.665 (6)
H12B	−0.013752	0.309075	0.350326	0.059*	0.665 (6)
C13	−0.1129 (5)	0.4046 (4)	0.1457 (8)	0.0532 (13)	0.665 (6)
H13A	−0.160649	0.392928	0.233262	0.064*	0.665 (6)
H13B	−0.117164	0.353052	0.072902	0.064*	0.665 (6)
C14	−0.1476 (9)	0.4885 (4)	0.0601 (12)	0.0487 (18)	0.665 (6)
H14A	−0.214573	0.476017	0.002703	0.058*	0.665 (6)
H14B	−0.094475	0.503776	−0.018419	0.058*	0.665 (6)
Cl1A	−0.1226 (6)	0.5970 (5)	0.1705 (9)	0.110 (2)	0.335 (6)
O3A	0.22328 (11)	0.38602 (10)	0.26936 (19)	0.0453 (3)	0.335 (6)
O4A	0.0068 (7)	0.4519 (9)	0.2390 (14)	0.085 (4)	0.335 (6)
C11A	0.1332 (8)	0.3392 (7)	0.3356 (18)	0.054 (4)	0.335 (6)
H11C	0.155056	0.306519	0.432005	0.064*	0.335 (6)
H11D	0.103552	0.295654	0.258975	0.064*	0.335 (6)
C12A	0.0522 (6)	0.4099 (7)	0.3732 (10)	0.074 (3)	0.335 (6)
H12C	−0.004449	0.381799	0.433809	0.089*	0.335 (6)
H12D	0.085853	0.456161	0.440578	0.089*	0.335 (6)
C13A	−0.0955 (10)	0.4210 (10)	0.199 (2)	0.074 (4)	0.335 (6)
H13C	−0.137940	0.416172	0.294477	0.089*	0.335 (6)
H13D	−0.091764	0.361186	0.149635	0.089*	0.335 (6)
C14A	−0.145 (2)	0.4874 (8)	0.086 (2)	0.074 (7)	0.335 (6)
H14C	−0.221538	0.475691	0.072880	0.089*	0.335 (6)
H14D	−0.112602	0.483019	−0.018129	0.089*	0.335 (6)

### Atomic displacement parameters (Å^2^)

**Table:**

	*U*^11^	*U*^22^	*U*^33^	*U*^12^	*U*^13^	*U*^23^
Br1	0.05356 (15)	0.06387 (18)	0.06366 (17)	0.01132 (11)	−0.02226 (11)	0.00047 (11)
O1	0.0518 (9)	0.0271 (7)	0.0773 (11)	−0.0023 (6)	0.0000 (8)	0.0077 (7)
O2	0.0477 (8)	0.0477 (8)	0.0536 (9)	0.0116 (7)	−0.0019 (7)	0.0146 (7)
N1	0.0340 (7)	0.0309 (8)	0.0368 (8)	0.0009 (6)	−0.0028 (6)	0.0022 (6)
C1	0.0325 (8)	0.0280 (8)	0.0319 (8)	0.0007 (6)	0.0024 (6)	0.0000 (6)
C2	0.0425 (10)	0.0245 (8)	0.0395 (9)	0.0003 (7)	−0.0007 (7)	−0.0035 (7)
C3	0.0445 (10)	0.0277 (8)	0.0418 (10)	0.0067 (7)	0.0006 (8)	0.0018 (7)
C4	0.0350 (9)	0.0396 (10)	0.0392 (9)	0.0051 (7)	−0.0035 (7)	0.0001 (8)
C5	0.0335 (8)	0.0312 (9)	0.0450 (10)	−0.0016 (7)	−0.0004 (7)	−0.0046 (7)
C6	0.0325 (8)	0.0245 (8)	0.0389 (9)	−0.0001 (6)	0.0023 (7)	0.0012 (7)
C7	0.0352 (9)	0.0280 (9)	0.0462 (10)	0.0007 (7)	0.0063 (7)	0.0044 (7)
C8	0.0359 (9)	0.0344 (9)	0.0392 (9)	0.0046 (7)	0.0051 (7)	0.0067 (7)
C9	0.0412 (10)	0.0429 (11)	0.0386 (10)	0.0024 (8)	−0.0072 (8)	−0.0078 (8)
C10	0.0411 (10)	0.0328 (9)	0.0575 (12)	−0.0031 (8)	−0.0088 (9)	−0.0027 (9)
Cl1	0.0912 (14)	0.0695 (10)	0.0751 (9)	0.0155 (10)	−0.0128 (9)	−0.0087 (7)
O3	0.0370 (7)	0.0433 (8)	0.0554 (9)	0.0009 (6)	−0.0002 (6)	0.0048 (7)
O4	0.0359 (19)	0.058 (2)	0.080 (2)	−0.0108 (15)	−0.0116 (14)	0.0262 (18)
C11	0.040 (2)	0.058 (3)	0.041 (2)	−0.0012 (19)	−0.0017 (15)	0.002 (2)
C12	0.0380 (16)	0.046 (2)	0.062 (2)	−0.0052 (13)	−0.0049 (14)	0.0137 (16)
C13	0.040 (2)	0.057 (3)	0.062 (3)	−0.0020 (19)	−0.010 (2)	0.007 (2)
C14	0.039 (3)	0.048 (3)	0.058 (3)	0.007 (2)	−0.009 (2)	0.004 (2)
Cl1A	0.113 (4)	0.077 (3)	0.138 (4)	0.023 (3)	−0.023 (3)	−0.047 (2)
O3A	0.0370 (7)	0.0433 (8)	0.0554 (9)	0.0009 (6)	−0.0002 (6)	0.0048 (7)
O4A	0.030 (3)	0.127 (9)	0.098 (7)	0.001 (5)	−0.001 (3)	0.064 (7)
C11A	0.059 (6)	0.046 (6)	0.056 (8)	0.010 (4)	0.012 (5)	0.016 (5)
C12A	0.051 (4)	0.097 (7)	0.075 (6)	0.015 (4)	0.016 (4)	0.027 (5)
C13A	0.061 (8)	0.074 (8)	0.087 (10)	−0.007 (6)	−0.009 (6)	0.027 (7)
C14A	0.052 (9)	0.102 (14)	0.068 (10)	−0.014 (8)	−0.007 (7)	0.006 (8)

### Geometric parameters (Å, º)

**Table:**

Br1—C4	1.8934 (19)	O3—C11	1.436 (5)
O1—C7	1.206 (2)	O4—C12	1.394 (5)
O2—C8	1.210 (2)	O4—C13	1.392 (5)
N1—C1	1.411 (2)	C11—H11A	0.9700
N1—C8	1.371 (2)	C11—H11B	0.9700
N1—C9	1.457 (2)	C11—C12	1.493 (6)
C1—C2	1.384 (2)	C12—H12A	0.9700
C1—C6	1.399 (2)	C12—H12B	0.9700
C2—H2	0.9300	C13—H13A	0.9700
C2—C3	1.392 (3)	C13—H13B	0.9700
C3—H3	0.9300	C13—C14	1.480 (6)
C3—C4	1.389 (3)	C14—H14A	0.9700
C4—C5	1.385 (3)	C14—H14B	0.9700
C5—H5	0.9300	Cl1A—C14A	1.773 (9)
C5—C6	1.384 (2)	O3A—C11A	1.438 (8)
C6—C7	1.463 (2)	O4A—C12A	1.387 (8)
C7—C8	1.558 (3)	O4A—C13A	1.385 (8)
C9—H9A	0.9700	C11A—H11C	0.9700
C9—H9B	0.9700	C11A—H11D	0.9700
C9—C10	1.508 (3)	C11A—C12A	1.485 (9)
C10—H10A	0.9700	C12A—H12C	0.9700
C10—H10B	0.9700	C12A—H12D	0.9700
C10—H10C	0.9700	C13A—H13C	0.9700
C10—H10D	0.9700	C13A—H13D	0.9700
C10—O3	1.415 (3)	C13A—C14A	1.481 (9)
C10—O3A	1.415 (3)	C14A—H14C	0.9700
Cl1—C14	1.772 (6)	C14A—H14D	0.9700
			
Br1···H12C^i^	3.05	O4···H14B^iii^	2.38
Cl1···O4	3.188 (5)	O4A···H14D^iii^	2.48
Cl1A···C11A^ii^	3.553 (4)	C1···C7^v^	3.542 (4)
Cl1A···O4A	2.720 (14)	C2···C10	3.537 (4)
Cl1···H9B^iii^	2.92	C5···C7^v^	3.356 (5)
Cl1A···H11D^ii^	2.99	C5···C8^v^	3.290 (2)
O1···C2^iv^	3.392 (4)	C6···C7^v^	3.251 (4)
O1···O2	2.951 (4)	C6···C6^v^	3.327 (2)
O1···C10^iv^	3.294 (5)	C8···C8^vi^	3.324 (4)
O1···C1^v^	3.397 (4)	C10···C2	3.537 (4)
O2···C8^vi^	3.093 (4)	C12A···O3	2.355 (5)
O2···N1^vi^	3.191 (2)	C2···H9A	2.89
O3···C5^v^	3.352 (5)	C4···H13C^i^	2.93
O3···C14^iii^	3.415 (4)	C5···H13C^i^	2.89
O3···O4	2.953 (4)	C9···H2	2.86
O3···N1	2.949 (5)	C10···H12A	2.99
O3A···C5^v^	3.352 (4)	C11···H5^v^	2.84
O3A···N1	2.949 (5)	H2···H9A	2.48
O3A···O4A	2.866 (4)	H2···H10B	2.59
O4···C14^iii^	3.302 (4)	H5···H12D^v^	2.58
O4A···C14A^iii^	3.37 (2)	H5···H11A^v^	2.41
O1···H2^iv^	2.47	H10A···H12A	2.52
O1···H9A^iv^	2.77	H10A···H11D	2.09
O2···H9B	2.60	H10B···H11B	2.35
O2···H3^iv^	2.85	H10B···H11C	2.34
O2···H14C^iii^	2.66	H12A···H13B	2.53
O2···H14A^iii^	2.50	H12B···H13A	2.39
O3···H5^v^	2.47	H12C···H13C	2.07
O3A···H5^v^	2.47	H14B···H14B^iii^	2.37
			
C1—N1—C9	124.25 (15)	O3—C11—C12	112.3 (4)
C8—N1—C1	110.85 (15)	H11A—C11—H11B	107.9
C8—N1—C9	124.61 (16)	C12—C11—H11A	109.2
C2—C1—N1	128.23 (16)	C12—C11—H11B	109.2
C2—C1—C6	120.95 (16)	O4—C12—C11	110.0 (4)
C6—C1—N1	110.81 (15)	O4—C12—H12A	109.7
C1—C2—H2	121.2	O4—C12—H12B	109.7
C1—C2—C3	117.63 (16)	C11—C12—H12A	109.7
C3—C2—H2	121.2	C11—C12—H12B	109.7
C2—C3—H3	119.5	H12A—C12—H12B	108.2
C4—C3—C2	121.00 (17)	O4—C13—H13A	109.4
C4—C3—H3	119.5	O4—C13—H13B	109.4
C3—C4—Br1	119.88 (14)	O4—C13—C14	111.1 (6)
C5—C4—Br1	118.47 (14)	H13A—C13—H13B	108.0
C5—C4—C3	121.64 (17)	C14—C13—H13A	109.4
C4—C5—H5	121.3	C14—C13—H13B	109.4
C6—C5—C4	117.33 (17)	Cl1—C14—H14A	108.7
C6—C5—H5	121.3	Cl1—C14—H14B	108.7
C1—C6—C7	107.34 (15)	C13—C14—Cl1	114.2 (6)
C5—C6—C1	121.43 (16)	C13—C14—H14A	108.7
C5—C6—C7	131.22 (17)	C13—C14—H14B	108.7
O1—C7—C6	130.79 (19)	H14A—C14—H14B	107.6
O1—C7—C8	124.15 (18)	C10—O3A—C11A	103.7 (4)
C6—C7—C8	105.05 (15)	C13A—O4A—C12A	113.9 (10)
O2—C8—N1	128.03 (19)	O3A—C11A—H11C	110.3
O2—C8—C7	126.21 (18)	O3A—C11A—H11D	110.3
N1—C8—C7	105.74 (15)	O3A—C11A—C12A	106.9 (7)
N1—C9—H9A	108.9	H11C—C11A—H11D	108.6
N1—C9—H9B	108.9	C12A—C11A—H11C	110.3
N1—C9—C10	113.45 (17)	C12A—C11A—H11D	110.3
H9A—C9—H9B	107.7	O4A—C12A—C11A	113.8 (10)
C10—C9—H9A	108.9	O4A—C12A—H12C	108.8
C10—C9—H9B	108.9	O4A—C12A—H12D	108.8
C9—C10—H10A	109.9	C11A—C12A—H12C	108.8
C9—C10—H10B	109.9	C11A—C12A—H12D	108.8
C9—C10—H10C	109.9	H12C—C12A—H12D	107.7
C9—C10—H10D	109.9	O4A—C13A—H13C	110.1
H10A—C10—H10B	108.3	O4A—C13A—H13D	110.1
H10C—C10—H10D	108.3	O4A—C13A—C14A	108.0 (12)
O3—C10—C9	109.14 (17)	H13C—C13A—H13D	108.4
O3—C10—H10A	109.9	C14A—C13A—H13C	110.1
O3—C10—H10B	109.9	C14A—C13A—H13D	110.1
O3A—C10—C9	109.14 (17)	Cl1A—C14A—H14C	110.5
O3A—C10—H10C	109.9	Cl1A—C14A—H14D	110.5
O3A—C10—H10D	109.9	C13A—C14A—Cl1A	106.2 (10)
C10—O3—C11	117.0 (3)	C13A—C14A—H14C	110.5
C13—O4—C12	117.0 (4)	C13A—C14A—H14D	110.5
O3—C11—H11A	109.2	H14C—C14A—H14D	108.7
O3—C11—H11B	109.2		
			
Br1—C4—C5—C6	178.48 (14)	C6—C1—C2—C3	0.9 (3)
O1—C7—C8—O2	2.9 (3)	C6—C7—C8—O2	−176.38 (18)
O1—C7—C8—N1	−178.03 (19)	C6—C7—C8—N1	2.66 (19)
N1—C1—C2—C3	−179.67 (18)	C8—N1—C1—C2	−174.80 (18)
N1—C1—C6—C5	178.66 (16)	C8—N1—C1—C6	4.7 (2)
N1—C1—C6—C7	−2.7 (2)	C8—N1—C9—C10	−109.2 (2)
N1—C9—C10—O3	67.1 (2)	C9—N1—C1—C2	−0.8 (3)
N1—C9—C10—O3A	67.1 (2)	C9—N1—C1—C6	178.69 (16)
C1—N1—C8—O2	174.61 (19)	C9—N1—C8—O2	0.7 (3)
C1—N1—C8—C7	−4.4 (2)	C9—N1—C8—C7	−178.36 (16)
C1—N1—C9—C10	77.7 (2)	C9—C10—O3—C11	−178.2 (3)
C1—C2—C3—C4	0.4 (3)	C9—C10—O3A—C11A	168.1 (7)
C1—C6—C7—O1	−179.2 (2)	C10—O3—C11—C12	−91.0 (5)
C1—C6—C7—C8	0.04 (19)	C10—O3A—C11A—C12A	−176.3 (8)
C2—C1—C6—C5	−1.8 (3)	O3—C11—C12—O4	−71.5 (6)
C2—C1—C6—C7	176.82 (16)	O4—C13—C14—Cl1	−69.5 (9)
C2—C3—C4—Br1	−179.34 (15)	C12—O4—C13—C14	−175.5 (6)
C2—C3—C4—C5	−0.8 (3)	C13—O4—C12—C11	−165.7 (5)
C3—C4—C5—C6	−0.1 (3)	O3A—C11A—C12A—O4A	67.7 (13)
C4—C5—C6—C1	1.4 (3)	O4A—C13A—C14A—Cl1A	−48 (2)
C4—C5—C6—C7	−176.87 (19)	C12A—O4A—C13A—C14A	164.5 (14)
C5—C6—C7—O1	−0.8 (4)	C13A—O4A—C12A—C11A	102.8 (13)
C5—C6—C7—C8	178.46 (19)		

Symmetry codes: (i) *x*+1, *y*, *z*; (ii) −*x*, *y*+1/2, −*z*+1/2; (iii) −*x*, −*y*+1, −*z*; (iv) −*x*+1, *y*+1/2, −*z*+1/2; (v) −*x*+1, −*y*+1, −*z*+1; (vi) −*x*+1, −*y*+1, −*z*.

### Hydrogen-bond geometry (Å, º)

**Table:**

*D*—H···*A*	*D*—H	H···*A*	*D*···*A*	*D*—H···*A*
C2—H2···O1^vii^	0.93	2.47	3.392 (4)	174
C5—H5···O3^v^	0.93	2.47	3.352 (4)	158
C14—H14*A*···O2^iii^	0.97	2.50	3.455 (5)	168
C14—H14*B*···O4^iii^	0.97	2.38	3.302 (6)	161

Symmetry codes: (iii) −*x*, −*y*+1, −*z*; (v) −*x*+1, −*y*+1, −*z*+1; (vii) −*x*+1, *y*−1/2, −*z*+1/2.

### Table 4. Comparison of the selected (X-ray and DFT) geometric data (Å, °).

**Table:**

Bonds/angles	X-ray	B3LYP/6-311G(d,p)
Br1—C4	1.8934 (19)	1.94411
Cl1—C14	1.772 (6)	1.88948
O3—C10	1.415 (3)	1.45191
O3—C11	1.436 (5)	1.45378
O2—C8	1.210 (2)	1.23688
O1—C7	1.206 (2)	1.23522
N1—C1	1.411 (2)	1.41761
N1—C8	1.371 (2)	1.39446
N1—C9	1.457 (2)	1.46194
C10—O3—C11	117.0 (3)	116.04457
C1—N1—C9	124.25(15	125.11525
C8—N1—C1	110.8 (2)	110.73581
C8—N1—C9	124.61 (16)	126.01387
C6—C1—N1	110.81 (15)	109.99463
C2—C1—N1	128.23 (16)	129.11525
C2—C1—C6	120.95 (16)	120.88664

### Table 5. Calculated energies.

**Table:**

Molecular Energy (a.u.) (eV)	Compound (I)
Total Energy *TE* (eV)	-106925,446
E_HOMO_ (eV)	-7,4517
E_LUMO_ (eV)	-0,9115
Gap *ΔE* (eV)	6,5402
Dipole moment *µ* (Debye)	7,9257
Ionisation potential *I* (eV)	7,4517
Electron affinity *A*	0,9115
Electro negativity *χ*	4,1816
Hardness *η*	3,2701
Electrophilicity index *ω*	2,6736
Softness *σ*	0,3058
Fraction of electron transferred *ΔN*	0,4301
